# Spike distributions of a population based hierarchical network of the rat amygdaloid complex

**DOI:** 10.1186/1471-2202-12-S1-P285

**Published:** 2011-07-18

**Authors:** Oliver Schmitt, Peter Eipert, Andreas Wree, Klaus-Peter Schmitz

**Affiliations:** 1 Department of Anatomy, University of Rostock, Germany; 2 Department of Biomedical Engineering, University of Rostock, Germany

## Background

The amygdaloid nuclear complex (ANC) is a conglomerate of gray matter lying in the depth of the anteromedial temporal lobe ventral to the lentiform nucleus. ANC is critical for producing appropriate emotional and behavioral responses to biologically relevant sensory stimuli. It constitutes an essential link between sensory and limbic areas of the cerebral cortex and subcortical brain regions, such as the hypothalamus, brainstem, and striatum, that are responsible for eliciting emotional and motivational responses. The connectome of the rat ANC is modeled in *neuroVIISAS* (a platform for mapping, visualization and analysis of connectivity data from tracing studies) that is used to generate realistic large scale networks of all regions of the central nervous system. The models are transferred by a *PyNEST* interface to the simulation engine *NEST* and simulation results can be analyzed and visualized by *neuroVIISAS*.

## Methods

700 Stereotactic tracing studies have been evaluated (metastudy) with regard to connection sources, targets and weights (density of a connection) to obtain a directed graph with weights of the ANC and further regions of the central nervous system (CNS) of the rat. *neuroVIISAS* is developed in *JAVA* using the *Java Development Kit* (JDK) (v1.6.0) and the development environment Eclipse (v3.2). The *Visualization Toolkit* (VTK) is used for 3D-visualization of brain region surfaces. The framework is used to analyze connectome at different levels of granularity by graph analytical methods. Based on results of local or region specific graph properties we are able to define spike injections in dependence of spatial properties of the region locations and their estimated total number of neurons. The definition of the network and simulation parameters is written into a *PyNEST* file that is processed by the simulation engine *NEST*. Simulation results are read by *neuroVIISAS* for analysis and visualization.

## Results

So far, the connectome of the bilateral rat CNS consists of 6690 regions and 39310 connections. 274 connections of 91 subregions of the ANC are found in 700 stereotactic tracing studies. The Internal connectivity of the ANC has an average cluster coefficient (ACC) of 0.285 (mean ACC of 1000 Barabasi-Albert (BA_1000_) network simulations: 0.084) and an average path length (APL) of 3.064 (APL-BA_1000_: 3.091) indicating a scale-free network. The posteromedial cortical nucleus has the highest internal in- (13) and outdegree (19), a low Shapley-rate of -0.287 and highest value for cyclic connections. The graph motif statistics of the internal ANC network revealed abundant divergence, chain and motifs with reciprocal connections (p ≤ 0.001 for BA_1000_). In dependence of the volume of the regions the number of neurons (6*10^5^) were estimated and downscaled by a factor of 1000 to allow simulation of leaky integrate and fire (LIF) neurons. 13069 LIF neurons (20% inhibitory, 80% excitatory) were generated and the simulations were performed for 100 ms with a spike injection of 5 ms within the PMCO. The number of connections between populations was correlated with the projection weights. Average firing frequencies of LIF neurons lie between 50 Hz and 475 Hz. The anterior basomedial nucleus and the medial amygdaloid nucleus anterodorsal part are first neighbors of PMCO and show the largest similarity (0.908) of their 0.3 ms binned spike distributions. Furthermore, in balanced randomizations of population internal connectivity as well as in small-world and scale free internal connection scheme, we observed oscillations that depend on the neighborhood distance to the spike injection region.

## Conclusions

Our data provide evidence for a topographic dependence of spike dynamics in multi-population models with different internal connection schemes. Furthermore, a time dependence of the spike distributions of populations having a larger distance to the spike injection populations produce delayed spike responses indicating a spatiotemporal consistency of the connectional ANC model.

